# Identification of Basement Membrane-Related Signatures in Gastric Cancer

**DOI:** 10.3390/diagnostics13111844

**Published:** 2023-05-25

**Authors:** Jinyun Wang, Dingwei Liu, Qixuan Wang, Yong Xie

**Affiliations:** 1 Department of Gastroenterology, Digestive Disease Hospital, The First Affiliated Hospital of Nanchang University, Nanchang 330006, China; 2 Queen Mary School, Medical College of Nanchang University, Nanchang 330006, China

**Keywords:** gastric cancer, basement membrane-related gene, prognostic, immune, chemotherapy response

## Abstract

Background: The basement membrane (BM) serves as a major barrier to impede tumor cell invasion and extravasation during metastasis. However, the associations between BM-related genes and GC remain unclear. Methods: RNA expression data and corresponding clinical information of STAD samples were downloaded from the TCGA database. We identified BM-related subtypes and constructed a BM-related gene prognostic model using lasso-Cox regression analysis. We also investigated the single-cell properties of prognostic-related genes and the TME characteristic, TMB status, and chemotherapy response in high- and low-risk groups. Finally, we verified our results in the GEPIA database and human tissue specimens. Results: A six-gene lasso *Cox* regression model (APOD, CAPN6, GPC3, PDK4, SLC7A2, SVEP1) was developed. Activated CD4+ T cells and follicular T cells were shown to infiltrate more widely in the low-risk group. The low-risk group harbored significantly higher TMB and better prognosis, favoring immunotherapy. Conclusions: We constructed a six-gene BM-related prognostic model for predicting GC prognosis, immune cell infiltration, TMB status, and chemotherapy response. This research provides new ideas for developing more effective individualized treatment of GC patients.

## 1. Introduction

The introduction is intended to briefly place the study in a broad context and highlight why gastric cancer (GC) is a significant global public health concern, with one million new cases annually, ranking as the fifth most commonly diagnosed malignant cancer worldwide. Additionally, it is the third leading cause of cancer-related death [1], despite the development of treatment strategies such as surgical excision and adjuvant chemotherapy. GC remains associated with an unfavorable long-term prognosis, leading to a significant health burden worldwide [2,3]. The rapid progression and metastasis of GC cells contribute to the poor prognosis of patient survival [4]. Therefore, a robust scoring system for prognosis and therapeutic response prediction is crucial for guiding therapeutic regimes and drug development.

The basement membrane (BM) is a fibrous matrix of ECM that underlies the endothelium of the vessel wall, composed mainly of glycoproteins, laminin, and type IV collagen secreted by the epithelial cells. The BM serves as a major barrier to impede tumor cell invasion and extravasation during metastasis [5]. The molecular and cellular mechanisms of metastasis involve several steps, including epithelial–mesenchymal transition (EMT), invasion, anoikis, angiogenesis, transport through vessels, and outgrowth of secondary tumors [6]. Previous studies have shown that BM-related genes regulate cell polarity, differentiation, migration, and survival, and that defects in these genes are closely related to cancer, diabetes, and fibrosis [7]. Therefore, BMs can serve as biomarkers to predict cancer progression and potential therapeutic targets. However, to date, the correlations between BM-related genes and GC have not been investigated comprehensively.

Here, we developed a six-gene BM-related prognostic model to predict GC prognosis, immune cell infiltration, tumor mutation burden (TMB) status, and chemotherapy response, providing new ideas for exploring more effective therapeutic approaches for GC patients. 

## 2. Materials and Methods

### 2.1. Data Acquisition and Processing

**Figure 1:**
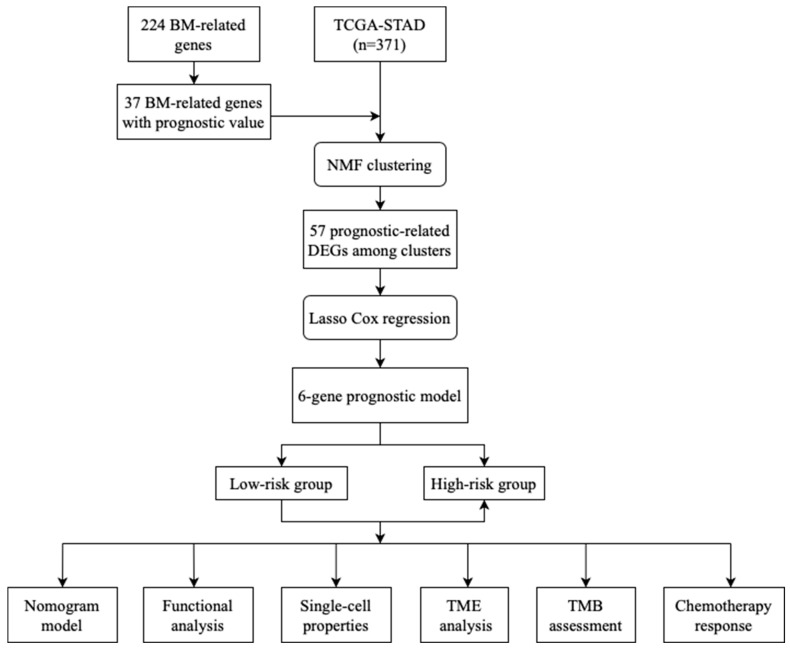
The flowchart of this study.

We retrieved the RNA expression data, mutation profiles, and the corresponding clinical information of GC samples from the TCGA database (TCGA-STAD). We excluded samples with incomplete survival data, resulting in a final sample size of 371. We collected 224 BM-related genes based on a previous study, including genes with confirmed protein BM zone localization, as well as those involved in protein interaction and protein-cleaving protease activity in the BM zone [7]. We extracted the expression levels of the 224 BM-related genes from each GC sample and then performed univariate Cox regression analysis to select BM-related genes associated with prognosis. Survival analysis was conducted using the “survival” R package. The flowchart of our study is presented in Figure 1. 

### 2.2. Using the NMF Algorithm to Identify Molecular Subtypes 

Using the non-negative matrix factorization (NMF) clustering algorithm to identify molecular subtypes, we analyzed the expression profile of 38 selected BM-related genes associated with prognosis for TCGA-STAD samples. We used the default “brunet” option and then performed 50 iterations, setting the number of clusters (K) as 2 to 10. We used the “NMF” R package to determine the average contour of the common member matrix, with the minimum member of each subclass being 10. The optimal clustering number was set to 3, as determined by cophenetic, dispersion, and silhouette indicators. Next, we identified differentially expressed genes (DEGs) among clusters using the “limma” R package to identify the BM-related genes differentially expressed between good and poor prognosis clusters (|log2 FC| > 1 and FDR < 0.05). 

### 2.3. Construction and Validation of the Prognostic Signature of BM-Related Genes

We divided the STAD samples into training and testing groups in a 7:3 ratio and performed univariate Cox regression on the training group to select prognosis-related genes from the 318 BM-related DEGs. We then used the identified 57 prognosis-related DEGs as features for developing the least absolute shrinkage and selection operator (LASSO) Cox regression analysis using the “glmnet” R package. Finally, we selected six genes associated with prognostic signatures. We calculated the risk score for each STAD sample using the formula Risk Score = ΣA*B, where A is the expression of each gene and B is the corresponding coefficient.

We used the “survival” and “survminer” R packages to plot Kaplan-Meier (KM) curves comparing the survival probability in high and low-risk groups, which were grouped by the median risk score. We plotted time-dependent receiver operating characteristic (ROC) curves using the “timeROC” R package. We used the STRING database (https://www.string-db.org/, accessed on 1 December 2022) and GeneMANIA (http://www.genemania.org, accessed on 1 December 2022) to develop a protein–protein interaction (PPI) network.

### 2.4. Functional Enrichment Analyses

We used the “limma” R package to obtain the DEGs between the high- and low-risk groups, ultimately identifying 231 DEGs. We performed GO and KEGG pathway enrichment analyses of these DEGs using the Metascape tool (https://metascape.org, accessed on 3 December 2022).

### 2.5. Nomogram Model Development and Assessment

To develop a prognostic nomogram to predict the 1-, 3-, and 5-year survival probability, we included age (>65 or ≤65), stage, and risk scores as variables, selected based on the results of uni- and multivariate Cox regression analysis. We used decision curve analysis (DCA) and clinical impact curves (CICs) to measure the net benefit of the nomogram in a clinical background. The nomogram model was developed and visualized using the “rms” and “replot” R packages.

### 2.6. Single-Cell RNA-Seq Analysis

We utilized the Tumor Immune Single-cell Hub (TISCH) database (http://tisch.compgenomics.org/, accessed on 5 December 2022), which integrates single-cell transcriptomic profiles across 27 cancer types by collecting high-quality data from 76 tumor databases. This enables the systematic comparison of gene expression between different cell types, tissues, patients, drug response groups, and cancer types [8]. We used the TISCH data to explore the distribution and expression of the six selected prognosis-related genes in the GSE134520 dataset, which were visualized using the Uniform Manifold Approximation and Projection (UMAP) plot. Furthermore, we also compared the expression level of these six genes in different immune cells between *Helicobacter. Pylori* (*H. pylori*) positive and negative samples.

### 2.7. Immune Microenvironment Analysis

To analyze the immune infiltration status and immunological functions of 22 immune cell subpopulations in STAD samples, we employed the cell type identification by estimating relative subsets of RNA transcripts (CIBERSORT) algorithm [9]. We used the ESTIMATE algorithm to generate immune and stromal scores, which allowed us to predict the tumor microenvironment status [10]. Furthermore, we evaluated the expression level of MHC molecules and immune checkpoints (ICPs) in the high- and low-risk groups to assess tumor progression and therapeutic response [11]. Additionally, we assessed the anticancer immune response and 36 common immune checkpoints in the low- and high-risk groups. 

### 2.8. Somatic Mutation Analysis

We downloaded the somatic mutation from the VarScan platform in the TCGA-STAD cohort. To visualize the somatic mutation frequency and distribution of variant genes in the low- and high-risk groups, we utilized the “maftools” R package. Furthermore, we evaluated the TMB as an indicator of the immunotherapy response and performed a correlation analysis of TMB and the prognostic signature.

### 2.9. Drug Sensitivity Prediction and Small Molecule Drug Identification

The Genomics of Drug Sensitivity in Cancer (GDSC) database (www.cancerRxgene.org, accessed on 12 December 2022) provides valuable information on drug sensitivity prediction in cancer cells and molecular markers of drug response [12]. To predict the drug response of TCGA-STAD, we used the “pRRophetic” R package, and the half-maximum inhibitory concentration (IC50) was estimated using Ridge’s regression. We utilized the Connectivity Map (cMap) database (https://www.broadinstitute.org/connectivity-map-cmap, accessed on 12 December 2022) to establish the connections between genes, targeting compounds, and diseases based on gene expression profiles. We screened the cMap database for potential therapeutic small-molecule drugs. Additionally, we submitted the DEGs (both upregulated and downregulated) between the high- and low-risk groups to the cMap database to screen for drugs that may improve GC progression. To obtain the 3D structures of candidate small molecules, we obtained data from the PubChem database (https://pubchem.ncbi.nlm.nih.gov/, accessed on 12 December 2022).

### 2.10. GEPIA Database Analysis

The GEPIA (Gene Expression Profiling Interactive Analysis) database (http://gepia.cancer-pku.cn/index.html, accessed on 17 December 2022) offers high-quality RNA sequencing expression data from the TCGA and GTEs database, enabling gene expression analysis between normal and tumor samples [13]. To identify genes with significant differences in expression between normal and GC patients, we utilized the six prognostic signatures in the GEPIA database.

### 2.11. RNA Extraction and Real-Time Quantitative Polymerase Chain Reaction (qPCR) 

The results of our GEPIA analysis showed that APOD and PDK4 had significantly different expression levels between normal and GC patients. To verify these results, GC and corresponding non-tumor normal tissue specimens were obtained from the pathology department of The First Affiliated Hospital of Nanchang University, with approval from the Ethics Committee on Medical Research (Ethical Application Ref: 2021006). All patients provided informed consent. We used the RNA extraction kit (Yeasen Biotechnology, Shanghai, China) to extract total RNA. cDNA synthesis was performed using the Hifair Ⅲ 1st Strand cDNA Synthesis SuperMix (Yeasen Biotechnology, Shanghai, China). qRT-PCR was performed using the SYBR Primix Ex Taq™ II (Tiangen Biotechnology, Beijing, China) on ABI-7500 instrument (Applied Biosystems, Waltham, MA, United States), with β-actin as the internal reference. The relative expression levels of the targeted genes were calculated using the 2^−△△Ct^ method, and the primers sequences are provided in Table S1.

### 2.12. HPA Database Analysis

The Human Protein Atlas (HPA) database (https://www.proteinatlas.org/, accessed on 8 May 2023) contains protein-level expression profiles and immunohistochemistry images of various cancer tissues. We used HPA databases to further confirm the expression levels of the selected six genes between GC and normal samples at protein levels.

### 2.13. Statistical Analysis

For continuous variables, the Student’s *t*-test was used, and for categorical variables, the Wilcoxon test was used. We performed univariate Cox regression analysis to determine the prognostic value of genes. Survival analysis was conducted using Kaplan-Meier analysis, and the significance was assessed by log-rank tests. The associations between two continuous variables were assessed using Spearman correlation analysis. *p* < 0.05 was considered statistically significant. All statistical analyses were performed using R software (4.1.2).

## 3. Results

### 3.1. BM-Related Molecular Subtype Identification in TCGA-STAD Patients

**Figure 2:**
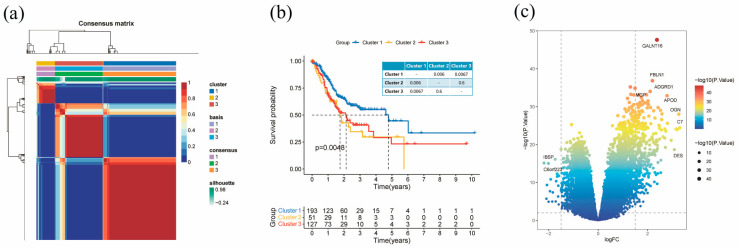
BM-related molecular subtype identification in TCGA-STAD patients. (**a**) Consensus map of NMF clustering (three clusters) (**b**) Survival curve of three clusters. (**c**) Volcano plot of DEGs.

We included 317 STAD samples from the TCGA database for our analysis. After conducting univariate Cox regression analysis on 224 BM-related genes, we selected 38 genes with prognostic values (Table S2). Based on the expression levels of these 38 BM-related genes from TCGA-STAD samples, we performed NMF clustering and divided the samples into three clusters (Figure 2a and Figure S1). Among these clusters, cluster 1 showed the best prognosis, while the prognoses of clusters 2 and 3 were relatively poor (Figure 2b). Therefore, we focused on identifying the DEGs between cluster 1 and clusters 2 and 3, which resulted in 318 DEGs with |log2 (fold change)| > 2 and *p* < 0.05 (Figure 2c and Table S3).

### 3.2. Prognostic Model Construction and Validation

**Figure 3:**
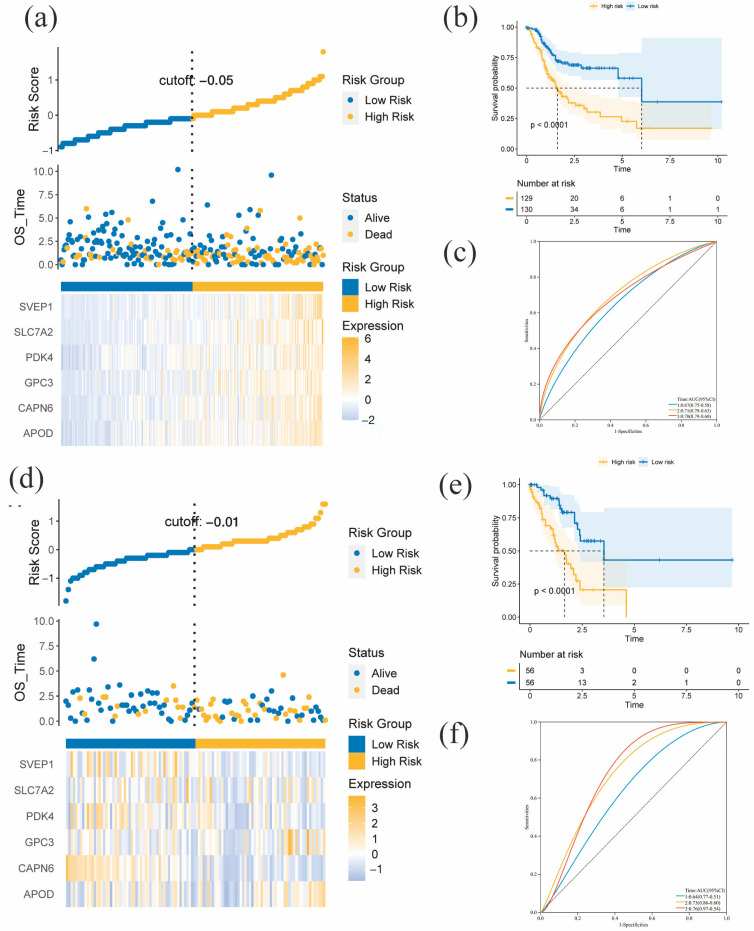
The expression levels of the six key genes and survival of the high- and low-risk groups in the training and testing cohort. (**a**,**d**) The distribution map of risk score, survival time, and heatmap (top to bottom) for the expression of six key genes in the training cohort (**a**) and validation cohort (**d**). (**b**,**e**) Kaplan-Meier curve of the high- and low-risk groups in the training cohort (**b**) and validation cohort (**e**). (**c**,**f**) ROC curve for predicting the 1-, 3-, and 5-year OS of TCGA-STAD patients in the training cohort (**c**) and validation cohort (**f**).

**Figure 4:**
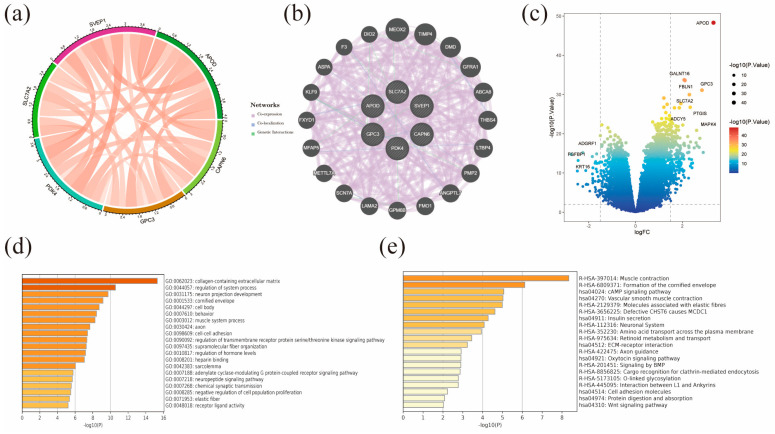
Correlation and enrichment analysis (**a**) Co-expression network of basement membrane-related genes. (**b**) Protein–protein interaction (PPI) network of six key prognostic genes. (**c**) Volcano plot of DEGs in the high- and low-risk groups. (**d**) GO analysis of DEGs. (**e**) KEGG analysis of DEGs.

After conducting univariate Cox regression analysis on the 318 DEGs, we screened 57 prognostic genes and included them in the lasso Cox regression model. Finally, we selected six genes (APOD, CAPN6, GPC3, PDK4, SLC7A2, and SVEP1) to construct the model. The formula used to calculate the risk scores of TCGA-STAD samples is as follows: APOD * (0.072687726146049) + CAPN6 * (−0.0154227752137368) + GPC3 * (0.0657545902748885) + PDK4 * (0.0258115254807578) + SLC7A2 * (0.101726534821853) + SVEP1 * (0.040030868592885). The median risk score in the training group was regarded as the cut-off value (−0.05) to classify TCGA-STAD samples into high- and low-risk groups. A high-risk score is identified as a factor for poor overall survival. The distribution of risk scores, overall survival time, and the expression heatmaps of the six genes in the high- and low-risk groups are shown in Figure 3c,f (Figure 3c for the training group and Figure 3f for the testing group). In addition, the Kaplan-Meier plots for high- and low-risk TCGA-STAD samples in the training and testing groups are shown in Figure 3d,e, respectively. The survival probability between the high- and low-risk groups was significantly different (*p* < 0.0001). We used time-dependent ROC to assess the model performance in predicting the 1-, 3-, and 5-year survival rates, shown in Figure 3e,h. The AUC of the training group’s 1-, 3-, and 5-year survival predictions were 0.67, 0.71, and 0.70. In the testing group, the AUC were 0.64, 0.73, and 0.76 for 1-, 3-, and 5-year survival prediction, respectively. Furthermore, we explored the interacting mechanisms between the six proteins; Figure 4a,b show intensive interactions and correlations among them.

### 3.3. Correlation and Enrichment Analysis 

The gene correlation analysis results in Figure 4a show a strong correlation between SVEP1 and PDK4 and APOD. The PPI network in Figure 4b indicated significant physical interactions between APOD, CAPN6, GPC3, PDK4, SLC7A2, and SVEP1 with MEOX2 and TIMP4. We identified 231 DEGs between the high- and low-risk groups, as shown in Figure 4c. GO enrichment analysis of the DEGs revealed their significant enrichment in the regulation of the system process and collagen-containing extracellular matrix, as demonstrated in Figure 4d. Furthermore, the KEGG analysis results indicated significant enrichment of DEGs in the cAMP signaling pathway, muscle contraction, and formation of the cornified envelope, as shown in Figure 4e.

### 3.4. Clinical Correlation Analysis Nomogram Model Construction

**Figure 5:**
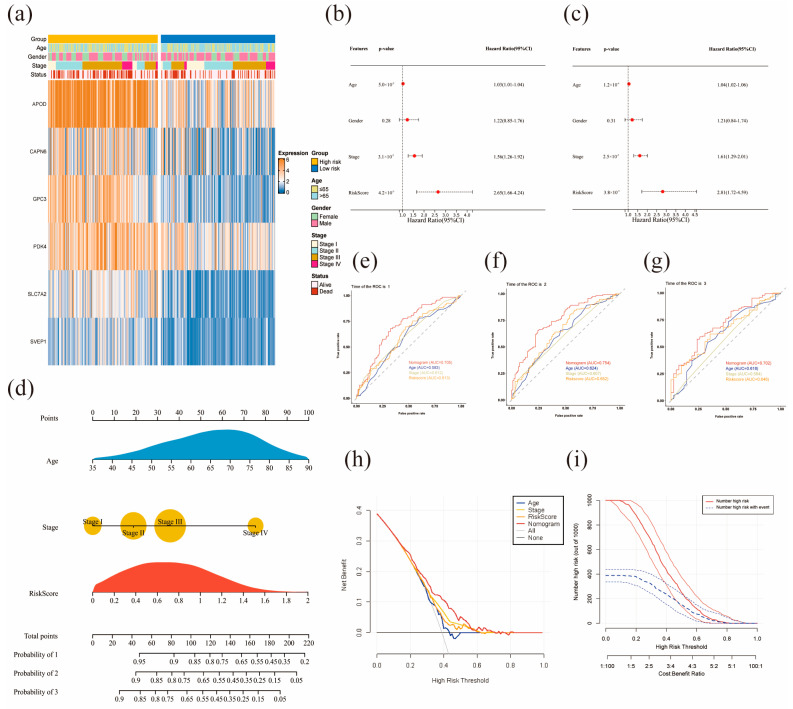
Associations between clinical factors, risk score and survival, and nomogram model construction: (**a**) the distribution of related clinical and genetic factors in the high- and low-risk groups. (**b**,**c**) The prognostic value of age, gender, stage, and risk score was identified by univariate (**b**,**c**) multivariate Cox regression. (**d**) The nomogram was used to predict patients’ 1-, 2-, and 3-year survival probability. (**e**–**g**) ROC curves of 1-, 2-, and 3-year survival prediction by age, stage, risk score, and nomogram. (**h**,**i**) Decision curve analysis (**h**) and clinical impact curve of the nomogram model (**i**).

Figure 5a provides an overview of the distribution of related clinical and genetic factors in the high- and low-risk groups. The results of uni- and multivariate Cox regression analyses, shown in Figure 5b,c, revealed a significant correlation between age, stage, risk score, and prognosis (*p* < 0.05). Of these factors, risk score was the most significant. However, sex was not found to have a significant association with prognosis. The results in Figure S4 show that the risk scores were significantly different in different age groups (>65 or ≤65) and stages (*p* < 0.05), and the survival time of patients in the high-risk group was significantly lower than that in the low-risk group (*p* < 0.05, Figure S4). We used the identified factors (age, stage, and risk score) to develop a nomogram model for predicting 1-, 3-, and 5-year survival probability. To evaluate the model’s net benefit, we used DCA and CIC analyses. We visualized the nomogram model using the “rms” and “replot” R packages.

### 3.5. Nomogram Model Construction and Assessment

We incorporated the significant prognostic factors, i.e., age, stage, and risk score, to build a nomogram model for predicting the 1-, 2-, and 3-year survival probability (Figure 5d). The total score for each patient can be calculated using the corresponding values for age, stage, and risk score. Each point corresponds to the 1-, 2-, and 3-year survival probability, enabling the prediction of GC patient prognosis. 

Next, we assessed the performance of the nomogram. Compared to using only age, stage, or risk score, the nomogram model demonstrated the best performance in predicting the survival probability of GC patients, having the highest AUC (Figure 5e–g, AUC: 0.705 for 1-year; 0.754 for 2-year; 0.702 for 3-year). The calibration curve of the nomogram model, presented in Figure S5, shows that the predicted survival probability aligned well with the actual outcome. The DCA result demonstrates that the nomogram model has the highest net benefit compared to other strategies, making it useful for clinical decision-making (Figure 5h). In addition, we used CIC analysis to assess the reliability of the nomogram model (Figure 5i). The red curve represents the number of patients classified as high risk at each threshold probability; the blue curve represents the number of classified high-risk patients with outcomes at each threshold probability. The results of CIC confirm that the nomogram model has a superior overall net benefit, indicating its significant predictive value.

### 3.6. Single-Cell Properties of the Six Prognostic-Related Genes 

**Figure 6:**
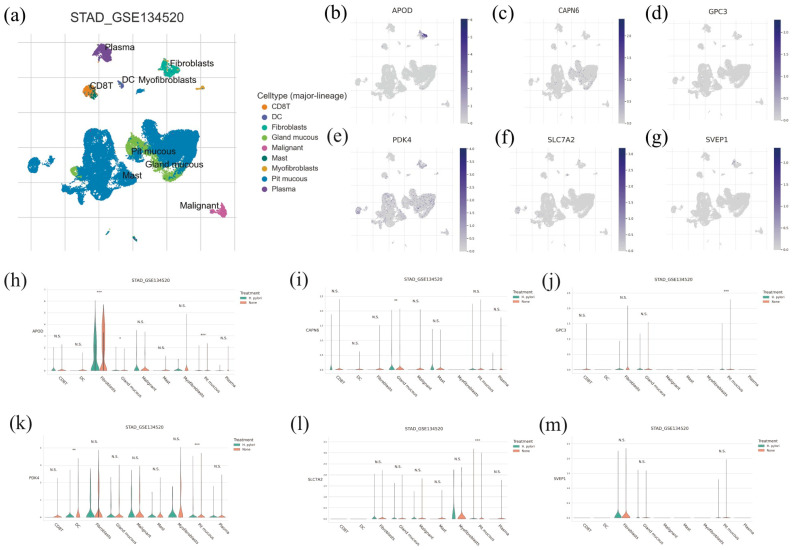
Single-cell properties of the six key genes. (**a**) The UMP plot shows a visualization of nine cell clusters of GSE134150. (**b**–**g**) The expression levels of APOD, CAPN6, GPC3, PDK4, SLC7A2, and SVEP1 in cell clusters. (**h**–**m**) The expression level of APOD, CAPN6, GPC3, PDK4, SLC7A2, and SVEP1 in H. pylori-positive TCGA-STAD samples and H. pylori-negative samples. N.S. not significant; * *p* < 0.05; ** *p* < 0.01; *** *p* < 0.001.

The UMP plot in Figure 6a shows a visualization of nine cell clusters, annotated as CD8 T cells, dendritic cells (DC), fibroblasts, gland mucous cells, malignant cells, mast cells, myofibroblasts, pit mucous cells, and plasma cells. Pit mucous cells and gland mucous cells comprise the majority of the cell populations. As shown in Figure 6b–g, APOD, PDK4, and SVEP1 mainly express in fibroblasts, whereas CAPN6, GPC3, PDK4, and SLC7A2 mainly express in pit mucous cells. The expression level of CAPN6 in gland mucous cells is also high. In addition, we assessed the expression level of the six genes in H. pylori-positive TCGA-STAD samples and H. pylori-negative samples (Figure 6h–m). The results demonstrate that the expression level of APOD expression in fibroblasts is significantly higher than that in H. pylori-positive TCGA-STAD samples. The expression of APOD and SLC7A2 in pit mucous cells also increases significantly in H. pylori-positive TCGA-STAD samples, while GPC3 and PDK4, it significantly decreases. CAPN6 expresses at a significantly lower level in gland mucous cells in H. pylori-positive TCGA-STAD samples, while APOD presents a higher level. Moreover, PDK4 presents a significantly lower level in the pit mucous cells and dendritic cells of H. pylori-negative TCGA-STAD samples. 

### 3.7. Tumor Immune Microenvironment Analysis

**Figure 7:**
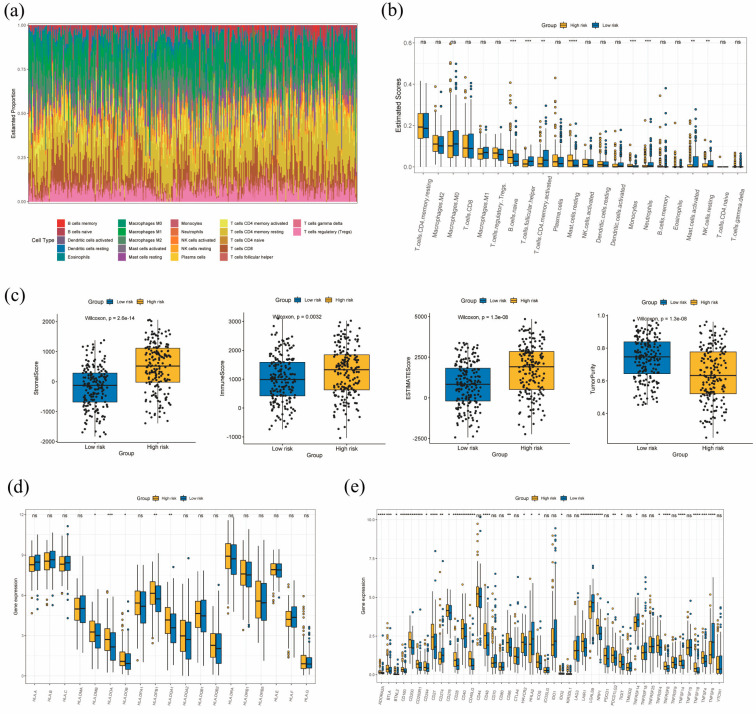
Tumor microenvironment properties of TCGA-STAD samples. (**a**) The proportion of different immune cells in stomach cancer samples. (**b**) The ESTIMATE scores of different immune cell subtypes in different risk groups. (**c**) The immune scores, stromal scores, ESTIMATE scores, and tumor purity levels in the high- and low-risk groups (**d**,**e**) The expression of (**d**) MHC molecules and (**e**) immune checkpoint-related genes in the high- and low-risk groups. ns. not significant; * *p* < 0.05; ** *p* < 0.01; *** *p* < 0.001; **** *p* < 0.0001.

Figure 7a shows the estimated proportion of immune cells in TCGA-STAD samples, indicating that macrophages and CD4 + T cells were the predominant immune cell types. The ESTIMATE scores of different immune cell subtypes in the high- and low-risk groups are shown in Figure 7b, revealing that mast cells, monocytes, resting NK cells, and naïve B cells were more abundant in the high-risk group, while activated CD4+ T cells and follicular T cells were more widely infiltrated in the low-risk group. To further investigate the TME patterns, we calculated stromal scores, immune scores, and ESTIMATE scores using the ESTIMATE algorithm. The results are displayed in Figure 7c. The scores were positively associated with higher risk and poorer prognosis, and the scores between the high- and low-risk groups were significantly different (*p* < 0.05). In contrast, the tumor purity scores were lower in the high-risk group. 

We also evaluated the gene expression level of 19 HLA family genes and 46 ICPs between the high- and low-risk groups (Figure 7d,e). The risk score was significantly associated with the expression of 5 HLA genes (HLA-DMB, HLA-DOA, HLA-DOB, HLA-DPB1, and HLA-DQA1), all of which were upregulated in the high-risk group. In addition, 31 ICPs displayed a significant difference between the high- and low-risk groups, among which 26 genes exhibited a higher level in the high-risk group, while 5 genes presented a lower level.

### 3.8. High-Risk Scores Are Negatively Correlated with High TMB

**Figure 8:**
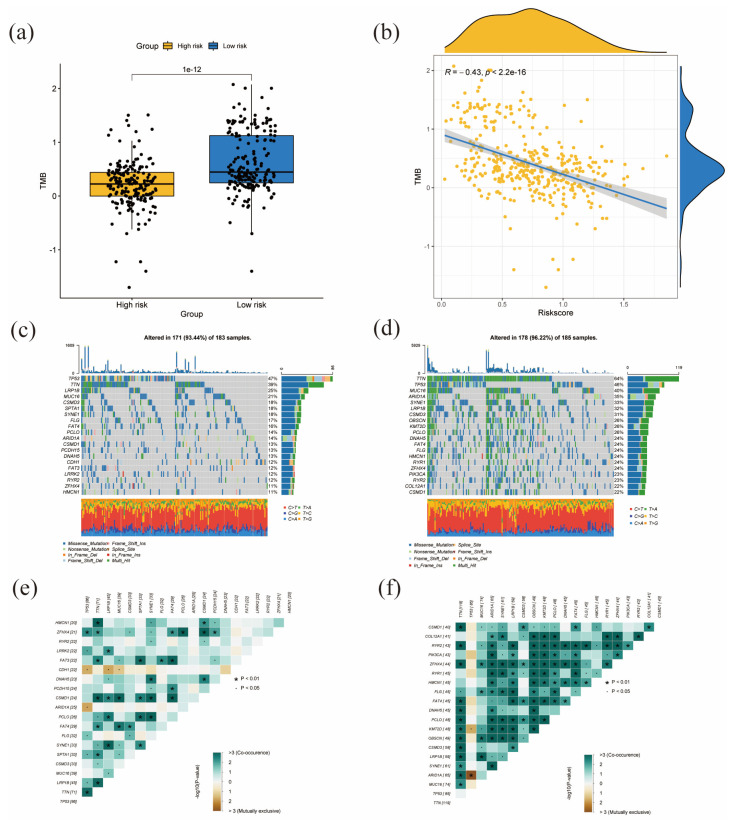
Associations between risk score and somatic mutation profiles in stomach cancer. (**a**) The difference of TMB in different risk groups. (**b**) The relationship between TMB and risk score. (**c**) The distribution of somatic mutation in the high-risk group. (**d**) The distribution of somatic mutation in the low-risk groups. (**e**) The different mutations of genes in the high-risk group. (**f**) The different mutations of genes in the low-risk groups.

We evaluated the TMB status of TCGA-STAD samples. As shown in Figure 8a, the low-risk group had significantly higher TMB. Figure 8b also shows that risk score was negatively correlated with TMB scores (R= −0.43, *p* < 0.05). Moreover, somatic mutations were presented in 93.44% (171/183) of samples in the high-risk group and 96.22% (178/185) of low-risk samples. The 20 genes with the highest mutation rates in the high- and low-risk groups are presented in the waterfall plot (Figure 8c,d). In the high-risk group, TP53 (47%), TTN (39%), LRP1B (25%), and MUC16 (21%) were the genes with the highest mutation rates (Figure 8c). Missense mutations were the most common variant type, and C > T mutations were in a large proportion of single nucleotide variations (SNVs). TTN (64%), TP53 (46%), and MUC16 (40%) were the most mutated genes in the low-risk group (Figure 8d). Missense mutations are more common in TTN and MUC16, and C > T also accounts for the vast majority of SNVs. Notably, the mutation frequency of TTN and MUC16 was higher in the low-risk group than in the high-risk group. In addition, multi-hit is also a commonly observed variant type in TTN and MUC16. Next, we assessed the correlations for the 20 mutated genes in the high- and low-risk groups, as shown in Figure 8e,f. Gene co-occurrence was much more commonly observed in the low-risk group.

### 3.9. Drug Response and Small Molecule Drug Prediction

**Figure 9:**
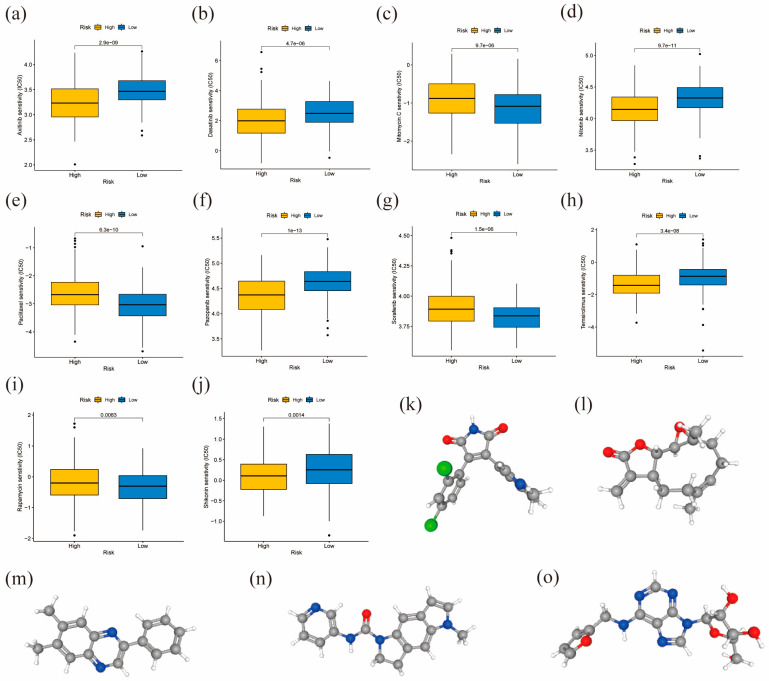
Chemotherapy drug sensitivity in high- and low-risk groups and small molecule drug prediction. (**a**–**j**) Drug sensitivity prediction of axitinib, dasatinib, mitomycin, nilotinib, paclitaxel, pazopanib, sorafenib, temsirolimus, rapamycin, and shikonin in the high- and low-risk groups. (**k**–**o**) Possible therapeutic agents for GC using the PubChem database: SB-216763, parthenolide, tyrphostin-AG-1295, SB-206553, and kinetin-riboside.

We assessed the drug responses for commonly used GC chemotherapy drugs in the high- and low-risk groups. Figure 9a–j demonstrates the differences in drug sensitivity between the two groups (*p* < 0.01), while the remaining results are listed in Figure S6. Our findings reveal that the high-risk group displayed greater sensitivity to axitinib (Figure 9a), dasatinib (Figure 9b), pazopanib (Figure 9f), and shikonin (Figure 9j). In addition, we predicted five possible therapeutic agents for GC using the cMap database and further visualized them using the PubChem database (Figure 9k–o). We conducted this analysis by examining the DEGs between the high- and low-risk groups.

### 3.10. Expression Verification of the Six Prognostic Signatures

**Figure 10:**
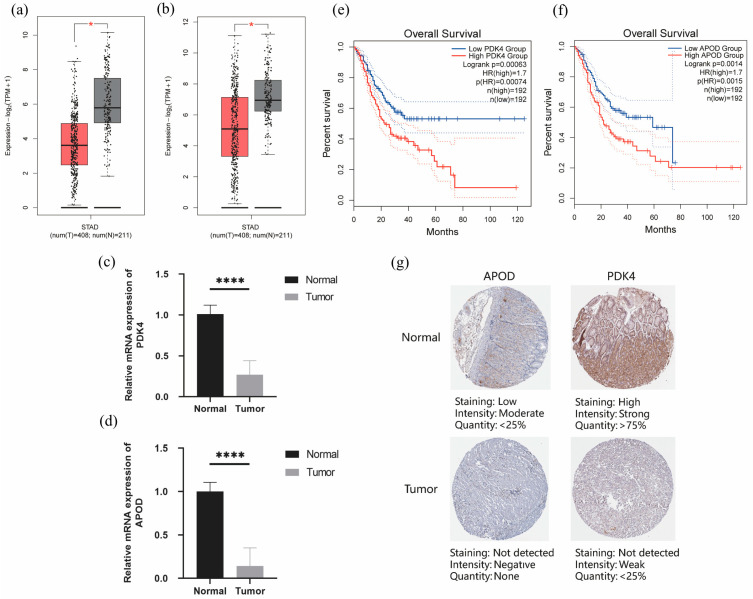
Two genes (PDK4 and APOD) in the prognostic model express significantly differently in normal and GC samples and are associated with survival. (**a**,**b**) The expression levels of PDK4 (**a**) and APOD (**b**) in normal and GC samples were derived from the GEPIA database. (**c**,**d**) The expression levels of PDK4 (**c**) and APOD (**d**) in 10 pairs of GC specimens and adjacent normal specimens. (**e**,**f**) Kaplan-Meier curve of GC patients with high and low APOD (**e**) and PDK4 (**f**) expression. (**g**) The HPA database revealed that both APOD and PDK4 exhibit lower protein expression levels in GC tissue. * *p* < 0.05, **** *p* < 0.0001; dot lines in (**e**,**f**) refer to 95% confidential interval.

To further explore the expression differences and prognostic value of the selected six key genes, we conducted further verification using the GEPIA and HPA databases. Our analysis of the GEPIA database revealed that PDK4 and APOD are significantly downregulated in GC samples (Figure 10a,b). We also validated the RNA expression of PDK4 and APOD in ten pairs of GC specimens and adjacent normal specimens and found that their expression levels were significantly higher in GC samples compared with the normal adjacent tissues (Figure 10c,d, *p* < 0.05). Moreover, the relatively high expression levels of PDK4 and APOD were associated with poor prognosis (Figure 10e,f, *p* < 0.05). Furthermore, our analysis of the HPA database reveals that both PDK4 and APOD exhibit lower protein expression levels in GC tissue (Figure 10g).

## 4. Discussion

GC is a major global public health concern, with a high incidence and mortality rate. [1]. Previous studies have suggested that BM-related genes conduct cell polarity, differentiation, migration, and survival, and that defects in these genes are closely related to cancer, diabetes, and fibrosis [7]. Thus, BMs can serve as biomarkers to predict cancer progression and potential therapeutic targets. However, to date, the correlations between BM-related genes and GC have not been investigated comprehensively. In this study, we developed a six-gene model to predict the prognosis of GC patients and then explored the functions of these genes in various aspects.

We utilized RNA expression data and clinical information from the TCGA database to cluster TCGA-STAD samples based on 38 BM-related genes with prognostic values. We identified 318 differentially expressed genes (DEGs) between cluster 1 with the best prognosis and clusters 2 and 3 with a relatively poor prognosis, among which 57 prognosis-related genes were selected. We developed a six-gene lasso Cox regression model based on these genes to predict GC patient survival. We also identified DEGs between high-risk and low-risk groups and performed a GO and KEGG pathway analysis. In addition, we incorporated the significant prognostic factors, i.e., age, stage, and risk score, to build a nomogram model. The DCA and CIC were adopted to evaluate the net benefit of the nomogram in a clinical setting. We also investigated the single-cell properties of the six prognostic-related genes, and the TME characteristic, TMB status, and chemotherapy response in the high- and low-risk group.

Our study developed a six-gene prognostic model (APOD, CAPN6, GPC3, PDK4, SLC7A2, and SVEP1) based on BM-related genes. This model can achieve comprehensive and accurate survival prediction of GC patients. It was reported that APOD was significantly downregulated in hepatocellular carcinoma, colorectal cancer, epithelial ovarian cancer, and breast cancer, and was closely associated with tumor progression and poor prognosis in these cancer types [14,15,16,17]. The increased expression level of CAPN6 can promote cell proliferation and play an anti-apoptotic role in cervical cancer and liver cancer cells, which favor tumor progression [18]. Han et al. demonstrated that GPC3 serves as a potential gene suppressing metastasis and can be used to predict the survival of GC patients [19]. PDK4 inhibits pyruvate oxidation, which is involved in the glucose-to-fatty acid metabolic switch [20]. Previous studies reported that PDK4 was decreased in different cancer types. PDK4 could restrain cell proliferation and trigger cell apoptosis in lung and breast cancer [21,22]. PDK4 was also found to be significantly downregulated in hepatocellular adenocarcinoma tissues; lower PDK4 levels in patients indicate lower overall survival rates and higher recurrence probability [23]. Additionally, PDK4 loss in ovarian cancer could activate the EMT and thus encourage cancer cell invasion and migration [24]. SLC7A2 encodes CAT2, which delivers arginine that activates mTORC1 to promote cancer cell growth responding to glutamine starvation. Arginine restriction could prevent GC cell migration in vitro [25,26,27]. SVEP1 encodes a multidomain cell adhesion protein mediating cell–cell adhesion in osteogenic cells [28]. SVEP1 was also shown to exhibit a significant association with poor prognosis of GC patients [29]. Notably, APOD and PDK4 are significantly downregulated in GC samples at RNA level, and the relatively high expression of APOD and PDK4 levels also indicates a poor prognosis. Therefore, BM-related genes are involved in the development of GC and might serve as potential markers for prognosis prediction.

Next, the TCGA-STAD samples were classified into high-risk and low-risk groups according to their risk scores, which were calculated using the lasso Cox regression prognostic model. We then identified the differentially expressed genes (DEGs) between the two groups. GO analysis revealed that DEGs were mainly enriched in the collagen-containing extracellular matrix. Previous studies showed that a dense collagen matrix increases the interactions between cells and ECM mediated by integrin, involving FAK and ERK phosphorylation in human gastric adenocarcinoma cells. This signaling pathway regulates GC cell proliferation and response to chemotherapy [30]. In addition, we integrated the significant prognostic factors, i.e., age, stage, and risk score, to build a nomogram model. DCA and CIC were adopted to evaluate the net benefit of the nomogram in a clinical setting. The nomogram model outperformed models that used age, stage, or risk score alone in predicting the survival probability of GC patients, as it had the highest AUC. Our studies thus provide a new scoring system for prognosis prediction in GC patients.

TME plays an essential role in tumor initiation, progression, metastasis, and tumor response to immunotherapies [31,32]. Therefore, understanding the TME in gastric cancer is crucial for predicting clinical outcomes and immunotherapeutic response. In this study, we used single-cell sequencing data to characterize the microenvironment of GC and investigated the properties of the six prognostic-related genes. Our results revealed that pit mucous cells and gland mucous cells account for most of the cell populations. APOD, PDK4, and SVEP1 mainly express in fibroblasts, while CAPN6, GPC3, PDK4, and SLC7A2 mainly express in pit mucous cells. TME components, like fibroblasts and immune cells, are correlated with tumor progression. Cancer-related fibroblasts (CAFs) are the major component in the stroma of many types of malignancies and contribute to tumorigenesis and progression. CAFs influence the occurrence and development of tumors through growth factor excretion, ECM remodification, angiogenesis promotion, and the suppression of anti-tumor immune response and are therefore associated with poor prognosis of GC [30]. The expression level of CAPN6 in gland mucous cells is also high. The surface mucus cells secrete mucus, covering the surface of the gastric mucosa to protect it from the acidic environment, which can also be regarded as a crucial factor in the TME of GC. Yu et al. showed that mucous cells exhibited the same trend as gastric cancer cells, i.e., oxidative stress response reduction, weak oxidative detoxification ability, and oxidative stress-induced cell death [33]. Furthermore, we assessed the expression levels of the six genes in H. pylori-positive TCGA-STAD samples and H. pylori-negative samples, since H. pylori contribute to a chronic inflammatory environment, which is considered a primary risk factor for GC progression [34].

To further understand the TME characteristics in GC patients and demonstrate the performance of our prognostic model in predicting the characteristics of TME, we used the ESTIMATE algorithm to quantify immune and stromal components in TCGA-STAD samples. We found that macrophages and CD4 + T cells account for a large proportion. Tumor-associated macrophages have been demonstrated to be associated with prognosis and resistance in GC treatment. Higher M2 macrophages in GC can induce an immunosuppressive GC phenotype [35]. Yuan et al. found that CD4+ T cells account for a significantly higher proportion of CD3+ cells in tumors compared with corresponding paracancerous tissue [35]. In addition, mast cell notably accounted for a larger proportion in the high-risk group, while activated CD4+ T cells and follicular T cells infiltrated more widely in the low-risk group. Previous studies showed that mast cell levels increase with tumor progression and can serve as an independent prognostic factor indicating reduced overall survival [36]. Zhong et al. demonstrated that mast cells may promote gastric cancer cell proliferation, migration, and invasion, as well as inhibiting apoptosis [37]. Here, the results demonstrate that all scores (stromal scores, immune scores, and ESTIMATE scores) are positively associated with higher risk and poorer prognosis; the scores between the high- and low-risk groups are significantly different. The high-risk group presents higher stromal, immune, and ESTIMATE scores. Previous studies suggest that stromal components, like fibroblasts, can affect antitumor immune responses and alter the tumor immunology [38]. The immune score was used to quantitate the abundance and phenotype of T cells [39]. The high-risk group was found to harbor higher immune scores and lower tumor purity levels, which showed that the high-risk group was strongly infiltrated by immune cells and had high immune activities. High immune activity or immune cell infiltration, which is associated with a worse prognosis. has been previously observed in other cancer types [40,41].

TMB is defined as the total number of mutations found in the genome of cancer cells. More mutations indicate a higher neo-antigen level, and thus, possibly also a higher probability of immunogenicity. Immunogenic neo-antigens are easier to recognize and eliminate by the immune system, thus triggering the activation of CD8+ cytotoxic T cells and T-cell-mediated antitumor activity [42,43]. Additionally, a higher TMB level indicates a better response to immunotherapy [44]. We evaluated the TMB status of TCGA-STAD samples and found that the low-risk group harbored significantly higher TMB and had better prognosis, favoring immunotherapy. Furthermore, risk scores are negatively correlated with TMB scores. These results show that the risk scores calculated by our prognostic model are associated with the TMB status of TCGA-STAD patients, so they can be used to predict the TMB status, prognosis, and therapeutic response to immunotherapy of GC patients. In addition, we evaluated the somatic mutation in TCGA-STAD samples. TP53, TTN, LRP1B, and MUC16 were the genes with the highest mutation frequencies in the high-risk group. TP53 mutates most frequently in GC [45]. Notably, the mutation frequencies of TTN and MUC16 were higher in the low-risk group than that in the high-risk group. Previous studies demonstrated that MUC16 mutation correlated with better prognosis and higher TMB in gastric cancer. TTN mutation has also been related to better immunotherapeutic response to ICP blockade in solid tumors [46,47]. In addition, Yang et al. reported that TTN and MUC16 show high potency in predicting TMB, which is in agreement with the results of our study [48].

Chemotherapy is the conventional treatment for advanced GC; it is used to relieve symptoms in patients with unresectable lesions and to reduce the chance of recurrence and metastasis in patients with localized lesions after resection. Although chemotherapy plays an essential role in treating local and metastatic GC, chemoresistance greatly limits chemotherapy response [36]. Therefore, patient stratification according to different chemotherapeutic responses might help refine the therapeutic regimes and thus maximize the effect of chemotherapy drugs. Our study demonstrated that the high-risk group presents more sensitivity to axitinib, dasatinib, pazopanib, and shikonin than the low-risk group.

After further exploration and experimental validation, our research may help guide future individualized treatments. Therefore, in the future, the risk score assessed by our prognostic model might be used to predict the chemotherapeutic sensitivity of GC patients.

## 5. Conclusions

We constructed a six-gene BM-related prognostic model for predicting GC prognosis, immune cell infiltration, TMB status, and chemotherapy response, providing new ideas for developing more effective individualized treatment for GC patients.

## Data Availability

The original contributions presented in the study are included in the article, further inquiries can be directed to the corresponding authors.

## Supplementary Materials

The following supporting information can be downloaded at https://www.mdpi.com/article/10.3390/diagnostics13111844/s1. Figure S1: NMF rank survey and survival probabilities between clusters; Figure S2: Results of lasso regression and the expression levels of the six key genes and survival of high- and low-risk groups in a total dataset; Figure S3: Protein-protein interaction network of the six key genes and GO analysis of 231 DEGs between the high- and low-risk groups; Figure S4: Risk scores and survival probabilities (male, female, age > 65, age < 65, stage I–II, and stage II–IV) between different genders, ages, stages groups; Figure S5: The calibration curve of the nomogram model; Figure S6: Drug sensitivity of Bortezomib, Bleomycin, Shikonin, Rapamycin, Gemcitabine, Etoposide between high- and low-risk group (*p* < 0.05); Table S1: The primers sequences of PDK4 and APOD for qPCR; Table S2: 38 genes with prognostic values selected from 224 BM-related genes; Table S3: 318 DEGs between cluster 1 and cluster 2 and 3; Additional file S1: Results of lasso-Cox model.
