# Supplementary figures and images for: Identification of Basement Membrane-Related Signatures in Gastric Cancer
Source: Diagnostics (Basel). 2023 May 25;13(11):1844. doi: 10.3390/diagnostics13111844 (PMC10252969; doi:10.3390/diagnostics13111844)

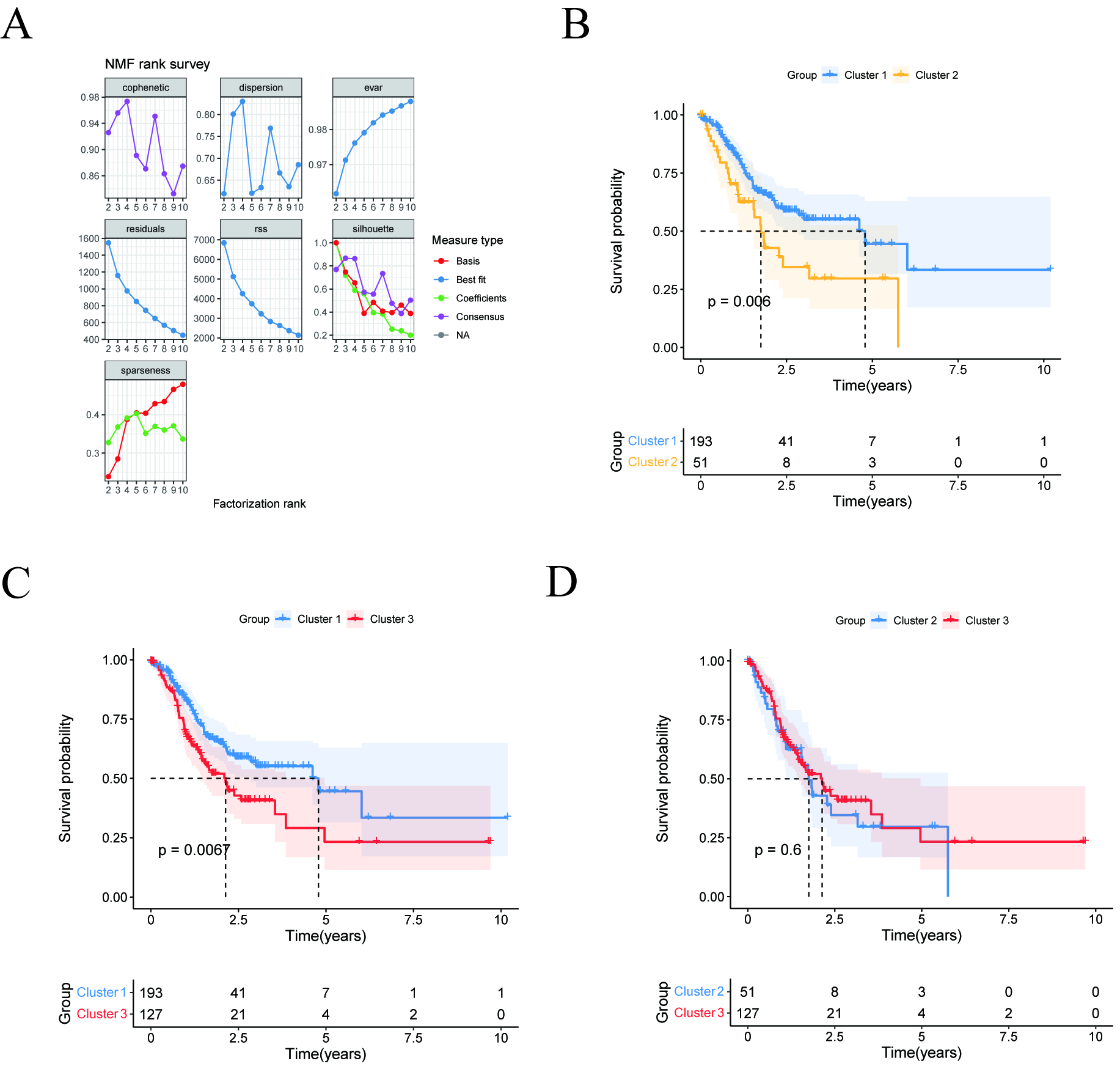

Supplement: Supplementary file 1 [file diagnostics-13-01844-s001.zip › Figure S1.tif]

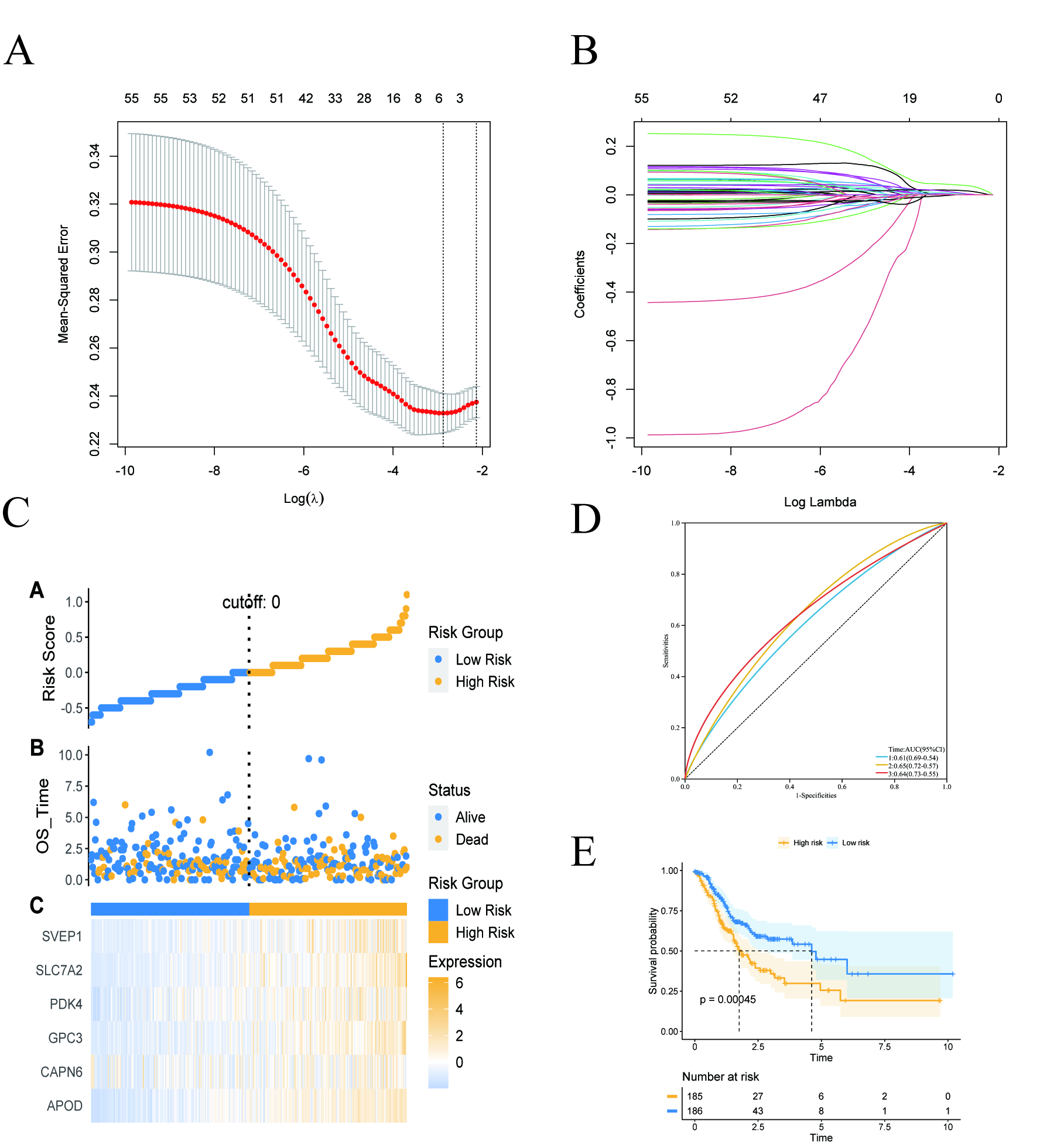

Supplement: Supplementary file 1 [file diagnostics-13-01844-s001.zip › Figure S2.tif]

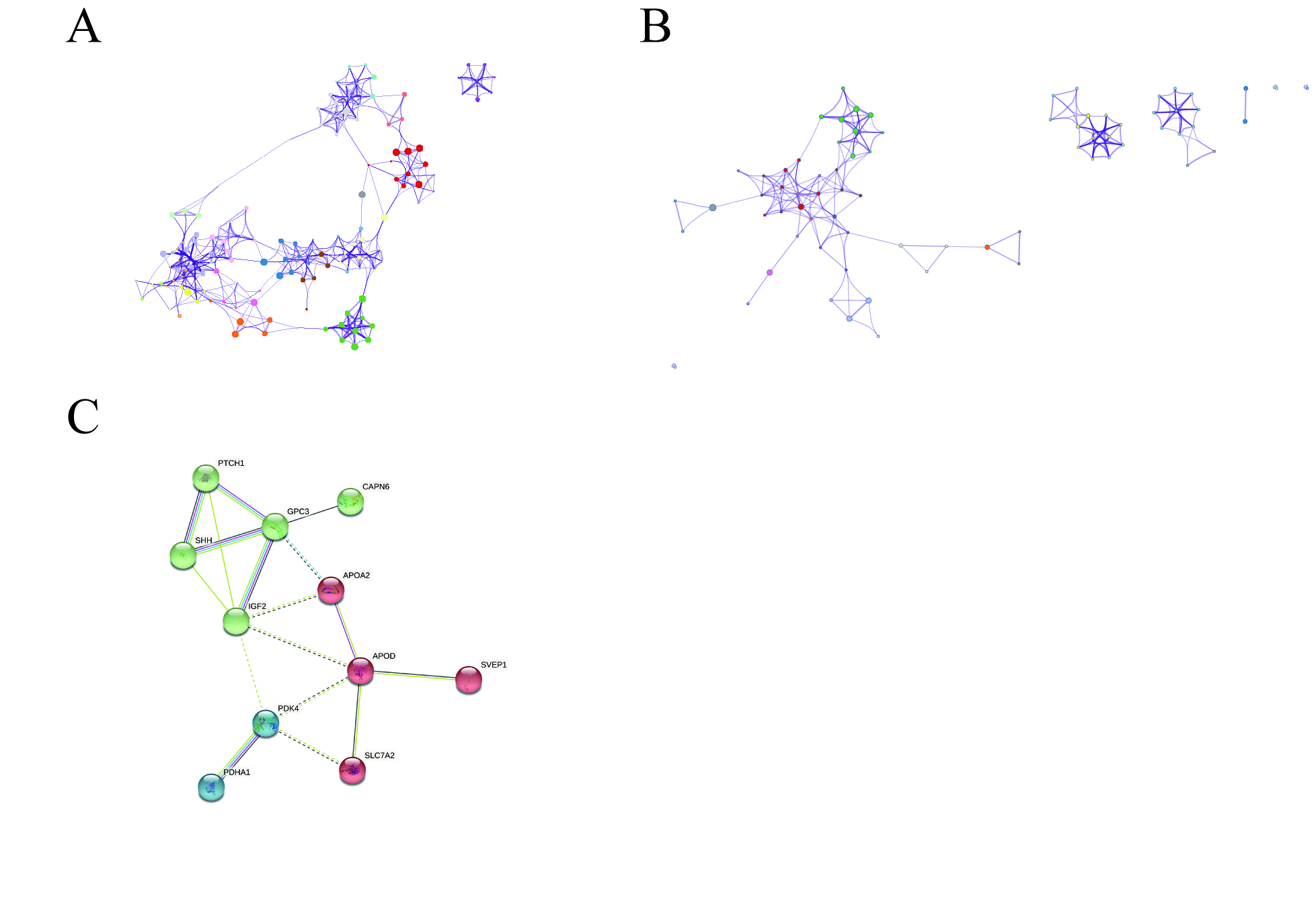

Supplement: Supplementary file 1 [file diagnostics-13-01844-s001.zip › Figure S3.tif]

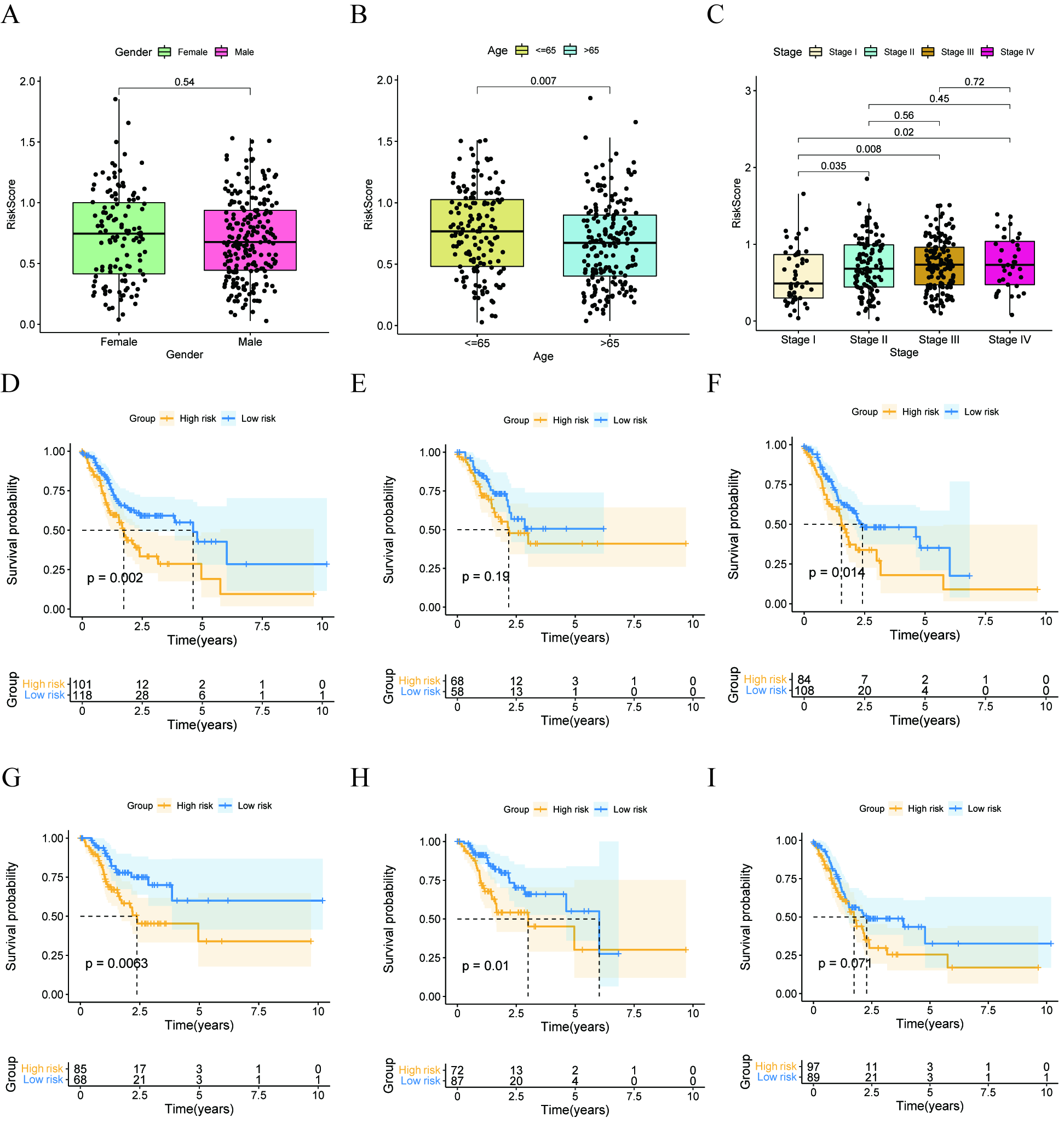

Supplement: Supplementary file 1 [file diagnostics-13-01844-s001.zip › Figure S4.tif]

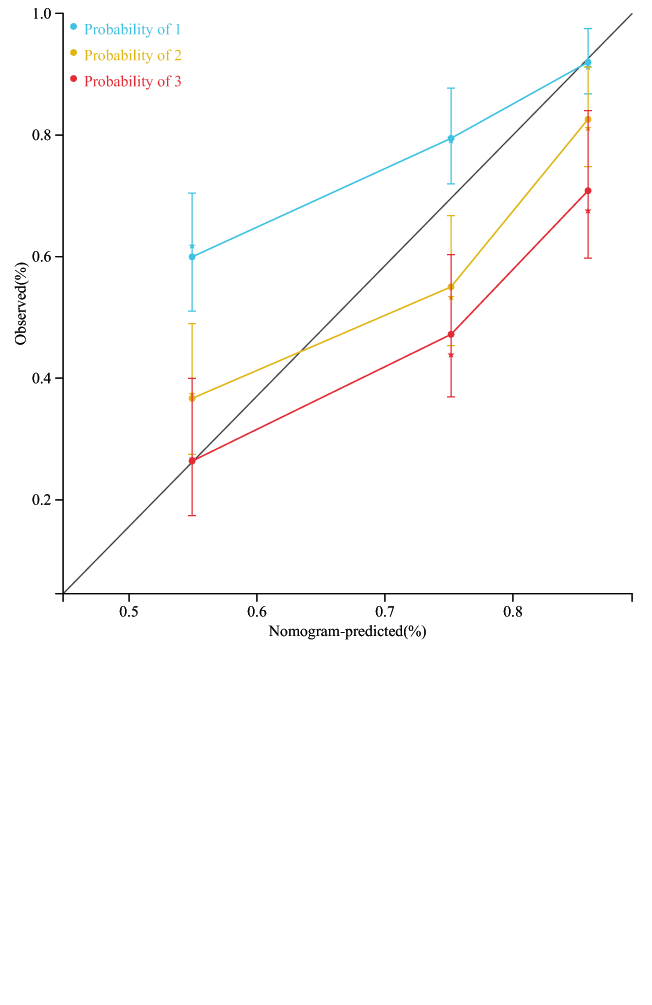

Supplement: Supplementary file 1 [file diagnostics-13-01844-s001.zip › Figure S5.tif]

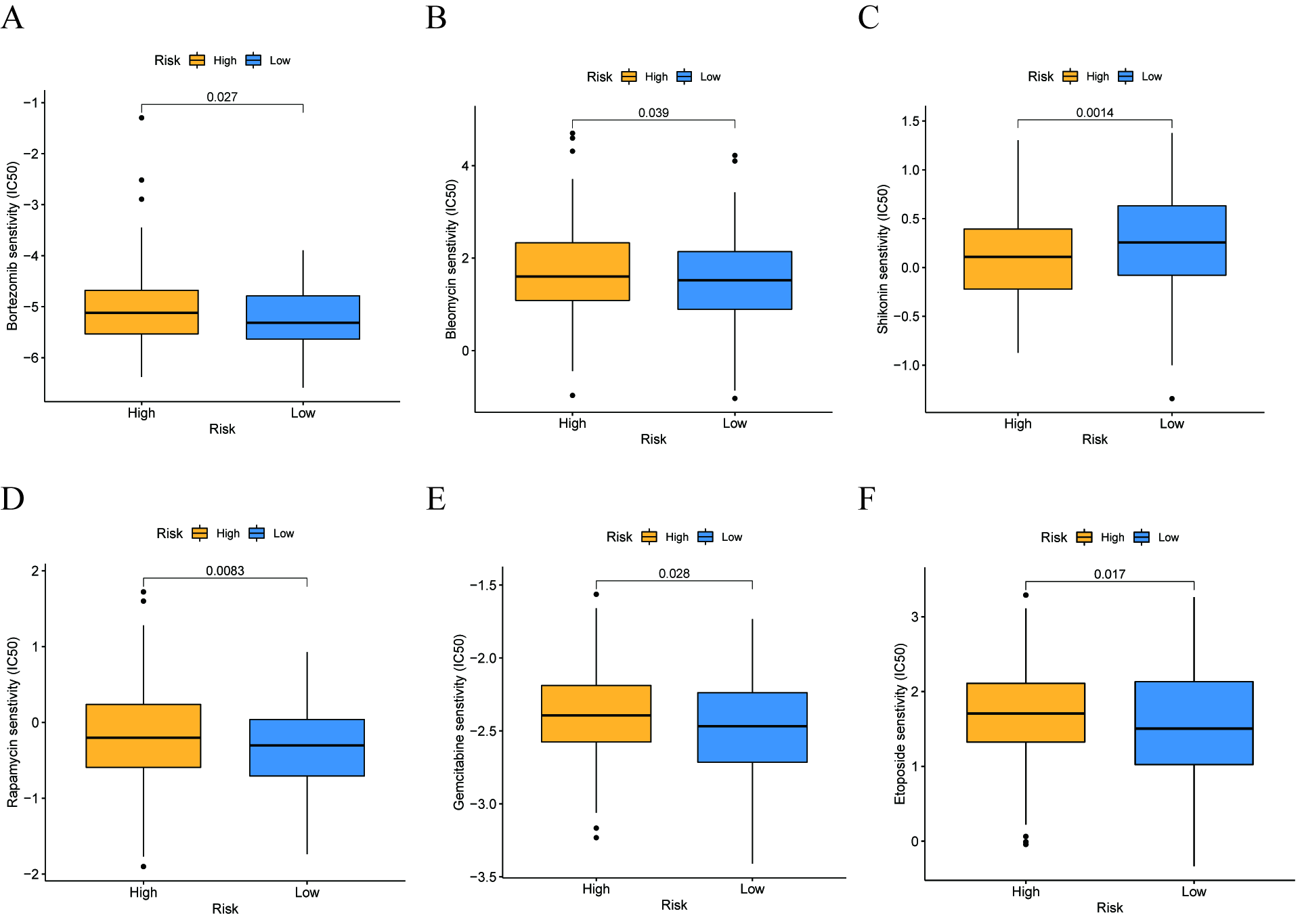

Supplement: Supplementary file 1 [file diagnostics-13-01844-s001.zip › Figure S6.tif]
